# Vaccination of metastatic renal cell carcinoma patients with autologous tumour-derived vitespen vaccine: clinical findings

**DOI:** 10.1038/sj.bjc.6604266

**Published:** 2008-03-25

**Authors:** E Jonasch, C Wood, P Tamboli, L C Pagliaro, S M Tu, J Kim, P Srivastava, C Perez, L Isakov, N Tannir

**Affiliations:** 1 Department of Genitourinary Medical Oncology, University of Texas MD Anderson Cancer Center, 1515 Holcombe Boulevard, Unit 1374, Houston, Texas 77030, USA; 2 Antigenics, 3 Forbes Road, Lexington, MA 02421, USA

**Keywords:** renal cell carcinoma, immunotherapy, autologous vaccine, phase II study

## Abstract

The aim of this study was to evaluate the clinical efficacy as determined by time to progression and response rate (RR) of autologous vitespen (formerly HSPPC-96; Oncophage, Antigenics Inc., New York, NY, USA) with and without interleukin-2 (IL-2; Proleukin: Chiron, Emoryville, CA, USA) in stage IV metastatic renal cell carcinoma (RCC) patients undergoing nephrectomy. Eighty-four patients were enrolled on study, and then underwent nephrectomy and harvest of tumour tissue for use in autologous vaccine manufacture. Initial treatment schedule started approximately 4 weeks after surgery and consisted of six injections: once weekly for 4 weeks, then two injections biweekly (vaccines administered at weeks 1, 2, 3, 4, 6, 8), followed by restaging at or around week 10. Patients who had stable or responsive disease continued to receive vaccine, with four more vaccinations biweekly (at weeks 10, 12, 14, 16). Patients who had progressive disease at week-10 evaluation received four consecutive 5-day-per-week courses of 11 × 10^6^ U of IL-2 subcutaneously (weeks 10, 11, 12, 13), with four doses of vitespen at 2-week intervals (at weeks 10, 12, 14, 16). At the next evaluation (week 18), patients with a complete response received two further cycles of vitespen (with IL-2 if also received during prior cycle) or until vaccine supply was exhausted. Patients with stable disease or partial response repeated their prior cycle of therapy. Disease progressors who had not yet received IL-2 began IL-2 treatment, and progressors who had already received IL-2 came off study. Of 60 evaluable patients, 2 demonstrated complete response (CR), 2 showed partial response (PR), 7 showed stable disease, and 33 patients progressed. Sixteen patients had unconfirmed stable disease. Two patients who progressed on vaccine alone experienced disease stabilisation when IL-2 was added. Treatment with vitespen did not result in a discernable benefit in the majority of patients with metastatic RCC treated in this study. Use in combination with immunoregulatory agents may enhance the efficacy of vitespen.

Metastatic renal cell carcinoma (RCC) has a poor prognosis, with a reported median survival of approximately 1 year (Motzer *et al*, 1999). Evidence that clear-cell RCC is an immunogenic tumour includes observed spontaneous tumour regression in 1–4% of patients (Elhilali *et al*, 2000), the presence of lymphocytic infiltrates in tumour, and responsiveness of RCC to cytokine therapy. A number of studies have been reported demonstrating the ability of interferon alpha and interleukin-2 (IL-2) to engender a tumour response (Fyfe *et al*, 1995; Pyrhonen *et al*, 1999; MRCRC Collaborators, 1999; Yang *et al*, 2003b; McDermott *et al*, 2005). High-dose IL-2 has consistently resulted in complete responses in 4–8% of patients, with durable responses in the majority of these individuals (Fyfe *et al*, 1995; Yang *et al*, 2003a; McDermott *et al*, 2005). In the case of interferon, two randomised studies demonstrated a modest survival advantage for those who received the agent (MRCRC Collaborators, 1999; Pyrhonen *et al*, 1999). A recent study demonstrated equal efficacy for standard and low dose interferon therapy (Tannir *et al*, 2006). Nevertheless, both interferon alpha and IL-2 are toxic, and the large majority of individuals who receive them derive no benefit. Efforts to develop more sophisticated immunotherapy platforms for this disease, including newer cytokine therapy (Alatrash *et al*, 2004), vaccine therapy (Uemura *et al*, 2006; Ernstoff *et al*, 2007), and nonmyeloablative transplantation (Childs *et al*, 2000; Artz *et al*, 2004; Rini *et al*, 2006), have been reported. Although these approaches have demonstrated tantalising indications of efficacy in a small group of individuals, they have not reached the threshold of therapeutic efficacy or safety.

Heat shock proteins (HSPs) are intra- and extracellular chaperones associated with stress response (Udono *et al*, 1994). Exogenous antigens chaperoned by a HSP can be channelled into the endogenous pathway, presented by MHC class I molecules, and recognised by CD8+ T lymphocytes (Suto and Srivastava, 1995). This process has been called cross-priming, and facilitates the recognition of tumour antigens if administered as an exogenous protein (Suto and Srivastava, 1995; Binder *et al*, 2001). Vitespen (formerly HSPPC-96; Oncophage; Antigenics, New York, NY, USA) is an autologous, tumour-derived HSP gp96-peptide complex that possesses potent-specific antitumour activity in melanoma and colon cancer xenograft models (Rivoltini *et al*, 2003), and demonstrated promising preliminary results in a phase I trial in colon cancer patients (Mazzaferro *et al*, 2003). Patients undergo tumour harvest, and vitespen is extracted, processed and administered as an injectable agent.

Owing to the documented response of RCC to immune therapy, it was considered an ideal disease for further investigation of vitespen. This paper is the first to present phase 2 data on patients with metastatic RCC receiving vitespen.

## PATIENTS AND METHODS

### Patients

Eligible patients had primary-intact renal tumour with bidimensionally measurable metastatic disease, and were scheduled to undergo nephrectomy at MD Anderson Cancer Center (MDACC). Eligibility criteria included the following: (1) pathological confirmation of RCC diagnosis; (2) ⩾9 g of non-necrotic, resectable primary RCC for vaccine preparation; (3) life expectancy of ⩾16 weeks; (4) Zubrod performance score of <2; (5) age of ⩾16 years; (6) normal white blood cell and platelet counts; (7) bilirubin <1.5 mg percent, AST <4 times the upper limit of normal, and serum creatinine of <2 mg percent.

All patients underwent clinical and radiographic staging before treatment. Patients were excluded from the study if they had received prior chemotherapy or immunotherapy; had active brain metastases, serious intercurrent medical illness requiring hospitalisation, history of primary or secondary immunodeficiency; were taking immunosuppressive drugs; were pregnant or lactating; or were on an intercurrent clinical trial. All patients gave written informed consent to participate in the study. The clinical protocol was approved by the Institutional Review Board of The University of Texas MD Anderson Cancer Center.

The study's primary efficacy end points were the rate of complete and partial response, the rate of secondary complete and partial response (after IL-2), and time to progression (TTP). Overall survival was also captured and recorded. Secondary end points included safety of vitespen treatment.

As of 6 July 1999, eligibility criteria were altered to include patients with a life expectancy of ⩾12 weeks. Patients with prior chemotherapy, immunotherapy, or radiotherapy were eligible but had to be fully recovered from prior therapy. Initial accrual of 60 patients was planned and subsequently increased to 79, with 84 patients ultimately enrolling at a single centre (MDACC).

### Vaccine preparation and administration

Patients underwent cytoreductive nephrectomy at MDACC. At least 9 g of fresh, non-necrotic tumour was acquired at the time of surgery and 3 g for immunological assays. Samples were placed into sterile vials, packed in dry ice, and shipped to Antigenics. At the Antigenics facility, tumour specimen was homogenised and centrifuged, and the resultant supernatant precipitated using ammonium persulphate. The resulting precipitate was then passed through sequential Sephacel columns, and the fractions containing vitespen were pooled, filtered, and stored at −80°C. Testing to assure product quality included sterility and endotoxin testing, and then aliquots were released to the MDACC research pharmacy for administration.

Initial treatment schedule started approximately 4 weeks after surgery and consisted of six injections: once weekly for 4 weeks, then two injections biweekly (vaccines administered at weeks 1, 2, 3, 4, 6, 8), followed by restaging at or around week 10. Patients who had stable or responsive disease continued to receive vaccine, with four more vaccinations biweekly (at weeks 10, 12, 14, 16). Patients who had progressive disease at week-10 evaluation received four consecutive 5-day-per-week courses of 11 × 10^6^ U of IL-2 subcutaneously (weeks 10, 11, 12, 13), with four doses of vitespen at 2-week intervals (at weeks 10, 12, 14, 16). At the next evaluation (week 18), patients with a complete response received two further cycles of vitespen (with IL-2 if also received during prior cycle) or until vaccine supply was exhausted. Patients with stable disease or partial response repeated their prior cycle of therapy. Disease progressors who had not yet received IL-2 began IL-2 treatment, and progressors who had already received IL-2 came off study.

### Response assessment

Lesions, measuring >1.0 cm, were considered measurable. Any lesion measuring ⩽1.0 cm was considered evaluable but not measurable. Bone lesions were considered measurable only if a measurable soft tissue component were present.

Complete response was defined as the disappearance of tumour for ⩾8 weeks. Partial response was defined as a ⩾50% decrease in the sum of the products of diameters of all measured lesions persisting for ⩾8 weeks, with no increase in size of lesion or appearance of new lesions. Minor response was defined as a decrease in the sum of the products of diameters of ⩾25% and <50% for ⩾8 weeks. Stable disease was defined as <25% growth or shrinkage for ⩾8 weeks. Progressive disease was defined as an increase of 25% in the sum of the products of diameters of any measurable lesion, or in estimated size of a nonmeasurable lesion or appearance of an unequivocal new lesion.

All radiological imaging evaluations performed on patients in this study were reviewed by an external independent radiologist for confirmation of data acquired from the study.

### Statistical considerations and methods

A patient was considered enrolled if the patient signed the informed consent form and met all eligibility criteria. An enrolled patient was considered treated if the patient received ⩾1 vitespen vaccine. A treated patient was considered evaluable for response assessment if the patient had a baseline visit and completed at least one post-treatment tumour assessment, or had a documented clinical progression in the absence of tumour assessment. All evaluable patients were included to assess primary efficacy. All treated patients were included to assess safety and secondary efficacy (overall survival).

In general, continuous variables were summarised as mean, s.d., median, minimum, and maximum. Categorical variables were summarised as the number and percentage of patients in each category. Time-to-event data were analysed using the product limit method (Kaplan–Meier estimate). All statistical analyses were performed using SAS®, version 8.2.

## RESULTS

Between July 1999 and March 2000, 84 patients were enrolled into the study. Of these, 60 were considered evaluable for response. Reasons for inevaluability included protocol ineligibility (*n*=12) and failure to receive vaccine (*n*=12). Of those who failed to receive vaccine, two were unresectable, one had no viable tumour tissue for vaccine production, three had non-RCC histology, and six died before receiving vaccine. Of these, two patients could not be weaned off the respirator post-nephrectomy. Two patients developed progressive brain metastases in the immediate postoperative period. Two patients had rapid progression of disease prior to nephrectomy and did not undergo surgery. Patient characteristics are outlined in Table 1.

Median time from enrollment to nephrectomy was 5.5 days (min–max 0–34 days), and median time from enrollment to first vaccine was 38 days (min–max 27–65 days).

Of the 60 evaluable patients, 39 patients had demonstrated clear progression by the time of first evaluation, and five patients demonstrated stable disease. Of the three patients who demonstrated partial response, two went on to show a complete response. Both complete responders demonstrated some degree of spontaneous tumour shrinkage postnephrectomy at baseline. The overall median TTP from the first vaccine administration of the vitespen only phase of the trial was 65 days (95% confidence interval (CI), 62.0–88.0 days) (Table 2a). By univariate analysis, performance status, tumour histology (conventional *vs* non-conventional), Memorial Sloan Kettering Cancer Center (MSKCC) risk factors, or number of metastatic sites did not impact RR or TTP (Table 3).

A total of 23 patients received vaccine plus IL-2 at the time of first progression. Of these patients, one demonstrated a PR and five had stable disease at the time of next evaluation. Overall TTP for this group was 168 days from the time of first treatment (including time on vitespen alone) (95% CI 122–233 days) (Table 2a).

**Figure 1:**
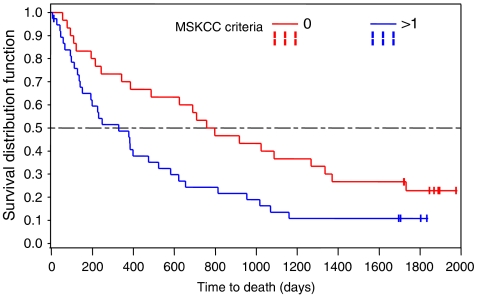
Overall survival by risk factors.

Median survival for all patients was 476 days (95% CI 249–691 days) (Table 2b). Survival was significantly impacted by the number of prognostic risk factors (*P*=0.017). Patients with 0 risk factors had a median survival of 779 days (95% CI 387–1338 days), whereas those with more than one risk factor had a survival of only 330 days (95% CI 153–525 days) (Figure 1).

Vitespen was not associated with frequent side effects. Only three patients experienced any treatment-related adverse events, and only one had any reported serious treatment-related side effects. The first patient reported some soreness at the injection site, and the second showed some flushing after vaccine administration. The patient who experienced reported serious treatment-related side effects was a 55-year-old male with RCC and metastases to the bone and lung. Immediately before his first vaccine, the patient had an elevated WBC count of 14.4. One, two, and three weeks later, the total WBC count had increased to 19.5, 18.1, and 30.6, respectively. One month after initiating treatment, the patient was seen in the clinic for fever, cough, and increasing shortness of breath, thought to be clinical signs of pneumonia. The patient reported that his fever and arthralgia seemed to occur during the period immediately following vaccine administration. The patient's symptoms also included somnolence, decreased urinary output and diffuse aches lasting up to 48 h after each vaccine injection. He was admitted to the hospital and a chest X-ray and blood cultures were obtained. Antibiotics (i.v.) were initiated. Chest X-ray showed a metastatic lesion and possible early pneumonia. CT scan of the brain showed no definite lesions. The investigator reported that he felt the patient experienced a paraneoplastic syndrome. The event was described as a leukemoid reaction with predominant eosinophilia, citing as a possible mechanism cytokine stimulation resulting in anuria, capillary leak syndrome, and oliguria. The patient's condition continued to deteriorate until he died in the hospital.

No responses were seen in patients with non-conventional histology, although one patient with conventional histology and a sarcomatoid component had a complete response. No association between Fuhrman nuclear grade and response was seen in patients with conventional RCC; one complete responder had Fuhrman nuclear grade II conventional type RCC, and the second complete responder had conventional type RCC with sarcomatoid dedifferentiation (Table 3).

Specific evaluation of those complete responders demonstrated three common features: good baseline performance status, lung only metastases, and some degree of spontaneous tumour regression post-nephrectomy. Both of these patients are in continuous complete remission over 7 years since the start of vitespen (Tables 4a and Tables 4b).

## DISCUSSION

Vaccine therapy has demonstrated promise in preclinical cancer models, although this promise has yet to translate into consistent clinical efficacy. The reasons for the failure to achieve clinical efficacy with vaccine therapy are unclear but a number of potential roadblocks exist. Common problems include the inability to engender antitumour immunity, which may due to tumour-induced anergy. Anergy may be induced directly through interaction of tumour cells with immune cells, or indirectly via production of humoral factors by tumours that locally block cytotoxicity, or via systemic factors, including vascular endothelial growth factor. Other difficulties include failure of effector cells to compete with growing tumour burden; antigen/MHC loss; and T-cell dysfunction or production of suppressor T cells, including regulatory T-cells. To optimise immunotherapy, correction of immune-deactivating signals and attenuation of inhibitory factors are likely necessary.

One of the specific impediments to engendering an immune response in cancer is the failure of exogenous antigen to induce a type I immune response. At the cellular level, priming of dendritic cells with exogenous antigen typically induces an MHC class II-mediated effect. Because CD8 cytotoxic T cells are thought to play a pivotal role in achieving immune-mediated tumour destruction, cross-presentation of exogenous antigen to the MHC class I pathway is an important event. Cross-presentation is facilitated by appropriate chaperone molecules, including HSPs.

Heat shock protein 90 in combination with tumour-specific antigens has been demonstrated to produce specific antitumour immunity (Srivastava and Das, 1984; Palladino *et al*, 1987; Blachere *et al*, 1993; Udono *et al*, 1994; Janetzki *et al*, 2000). The clinical trial being reported in this paper was initiated in 1999 to help address the issue of whether treating MRCC patients with the autologous HSP90 product vitespen induces an antitumour response, and whether subcutaneous outpatient IL-2 can convert vitespen nonresponders into responders.

As seen in Table 1, evaluable individuals enrolled in this trial had good performance status, with 31 (52%) exhibiting a baseline performance status of 0. Forty-five patients had only 1 or 2 sites of disease, and 48 had conventional type RCC. Twenty-five patients had MSKCC good-risk prognosis after nephrectomy. Overall, this group of individuals is expected to have a favourable outcome, and to derive the greatest benefit from immunotherapy. It is important to note, however, that all of these individuals presented with metastatic disease upfront, and the time to first treatment was by definition less than 1 year. Therefore, using the criteria published in 2002, all patients had at least one negative risk factor. In addition, it is apparent that a number of individuals had sufficiently rapid disease progression to prevent nephrectomy or vaccine administration. The absence of upfront systemic therapy may be in part responsible for this rapid progression despite good initial patient characteristics.

The vaccine was well tolerated in the vast majority of patients, and this is consistent with the data accumulated in 771 patients treated to date with Oncophage, where no other related paraneoplastic synderomes were reported (Antigenics, Oncophage Investigators Brochure). Time to progression from the first vaccine administration for vaccine alone was 65 days (Table 2a). It should be noted that it took a median of 38 days from the enrolment date to the first vaccine treatment. Adding IL-2 did not appear to have a significant impact on clinical outcome. Median overall survival was 476 days (15.6 months), in keeping with the reported overall survival for patients with good and intermediate prognosis metastatic RCC. Although these data suggest that patients were not harmed by receiving vitespen, the relatively short TTP suggests minimal efficacy in the majority of individuals on study.

The two individuals who ultimately achieved a complete response showed signs of spontaneous regression after surgery and before starting vitespen. It is possible that the complete response seen in these agents was solely as a result of host factors and was not due to the study drug. On the other hand, since a number of individuals who show spontaneous regression ultimately develop progressive disease without therapy, it is possible that the study drug may have improved the durability of response in these two individuals. It is important to note that one of these patients had sarcomatoid histology, not traditionally associated with immune responsiveness.

Several studies have been published in the past 10 years looking at the efficacy of interferon (MRCRC Collaborators, 1999; Pyrhonen *et al*, 1999), IL-2 (Yang *et al*, 2003b; McDermott *et al*, 2005; Tannir *et al*, 2006), and antivascular-targeted therapies (Hainsworth *et al*, 2005; Escudier *et al*, 2007; Motzer *et al*, 2007) in the treatment of patients with metastatic RCC. These treatments demonstrate incremental gains in survival (MRCRC Collaborators, 1999; Pyrhonen *et al*, 1999), a small but consistent and durable complete response rate (Yang *et al*, 2003b; McDermott *et al*, 2005) or prolonged progression-free survival rates (Hainsworth *et al*, 2005; Escudier *et al*, 2007; Motzer *et al*, 2007). From a clinical perspective, vitespen does not reach the threshold of therapy when compared to these other agents.

It is not clear why vitespen failed to provide the anticipated clinical benefit. Possibilities include an inherently weak immune effect achieved by the proposed mechanism of action of vitespen, or the inability to overcome T-regulatory cell inhibition. Other possibilities include an inappropriate route or schedule of vaccine administration. It is also possible that other forms of immune costimulation or adjuvants are required to optimise vitespen's clinical effect. The combination of vitespen with anti-CTLA-4 blockade may also improve vitespen's efficacy.

In conclusion, this study shows that vitespen is a relatively ineffective agent in patients with metastatic RCC. If immune modulation has occurred in these individuals, it is at a subclinical level. Future investigations will require combination of vitespen with immunostimulatory or targeted agents that may permit this agent to reach the threshold of consistent clinical efficacy in the metastatic setting.

## Figures and Tables

**Table 1:** Patient characteristics

**Variable**	***N*=60 (%)**
Median age in years (range)	57.0 (39.0–79.0)
	
*Gender*	
Male	47 (78)
Female	13 (22)
	
*Zubrod performance status*	
0	31 (52)
1	19 (32)
2	8 (13)
Missing PS	2 (3)
	
*Tumour pathology*	
Conventional grade II	10
Grade III	23
Grade IV	15
	
*Sarcomatoid features*	7
Other	5
	
*No. of disease sites*	
1	18
2	28
3	13
4	2
	
*MSKCC risk groups*	
0	25
1–2	30
3 or greater	2
Missing data	3

**Table 2a:** Response evaluation according to treatment

	**Overall**	**HSPCC alone**	**HSPCC+IL-2**
**Response rate**	***N*=60 (%)**	***N*=59 (%)**	***N*=23 (%)**
CR	2 (3.3)	2 (3.4)	0
PR	2 (3.3)	1 (1.7)	1 (4.3)
SD	7 (11.7)	5 (8.5)	5 (21.7)
PD	33 (55.0)	39 (66.1)	14 (60.9)
Unconfirmed stable disease	16 (26.7)	12 (20.3)	3 (13.0)
TTP from first treatment in days	65 (95% CI 63.0–88.0)	65 (95% CI 62.0–88.0)	168.0 (95% CI 122.0–233.0)

**Table 2b:** Overall survival

	**Overall**	**HSPCC Alone**	**HSPCC + IL-2**
***N***	**72**	**35**	**37**
Survival from first treatment in days	476 (95% CI 249.0–691.0)	584 (95% CI 123.0–1026.0)	476 (95% CI 330.0–691.0)

**Table 3:** Response according to patient characteristics

**Parameter**	**Median time to progression (days)**	**95% Confidence interval**
*Performance status*		
0	65.0	(61.0–91.0)
1 or greater	70.0	(63.0–118.0) (NS)
		
*MSKCC risk factors*		
0	69.0	(63.0–118.0)
2 or greater	64.0	(61.0–77.0) (NS)
		
*Tumour histology*		
Conventional	69.0	(62.0–91.0)
Nonconventional or sarcomatoid	64.0	(62.0–93.0) (NS)
		
*Number of metastatic sites*		
Single	76.0	(60.0–117.0)
Multiple	65.0	(62.0–91.0) (NS)

**Table 4a:** Related^a^ adverse events reported among treated patients (*N*=72)^b^

**Number of patients with ⩾1 related adverse event (*n*(%))**	**3 (4.2)^c^**
**Adverse event**	***n* (%)**
*Blood and lymphatic system disorders*	
Anaemia	1 (1.4)
Leukocytosis	1 (1.4)
	
*Eye disorders*	
Pupils unequal	1 (1.4)
	
*General disorders and administration site conditions*	
Pyrexia	1 (1.4)
	
*Metabolism and nutrition disorders*	
Metabolic acidosis	1 (1.4)
	
*Musculoskeletal and connective tissue disorders*	
Musculoskeletal discomfort	1 (1.4)
	
*Neoplasms benign, malignant, and unspecified*	
Paraneoplastic syndrome	1 (1.4)
	
*Psychiatric disorders*	
Mental status changes	1 (1.4)
	
*Renal and urinary disorders*	
Renal failure, acute	1 (1.4)
	
*Respiratory, thoracic, and mediastinal disorders*	
Capillary leak syndrome	1 (1.4)
Dyspnoea	1 (1.4)
Respiratory failure	1 (1.4)
	
*Vascular disorders*	
Flushing	1 (1.4)

a Adverse events assessed by the principal investigator as possibly, probably, or highly probably related to HSPPC-96 administration.

b Patients are counted once within each body system and preferred term.

c All three patients were in the treatment group that received only HSPPC-96.

**Table 4b:** Severe related^a^ adverse events reported among treated patients (*N*=72)^b^

**Number of patients with ⩾1 severe related adverse event**	**1 (1.4)^c^**
**Adverse event**	***n* (%)**
*Blood and lymphatic system disorders*	
Anaemia	1 (1.4)
	
*Eye disorders*	
Pupils unequal	1 (1.4)
	
*Metabolism and nutrition disorders*	
Metabolic acidosis	1 (1.4)
	
*Neoplasms benign, malignant, and unspecified*	
Paraneoplastic syndrome	1 (1.4)
	
*Psychiatric disorders*	
Mental status changes	1 (1.4)
	
*Renal and urinary disorders*	
Renal failure, acute	1 (1.4)
	
*Respiratory, thoracic, and mediastinal disorders*	
Capillary leak syndrome	1 (1.4)
Dyspnoea	1 (1.4)
Respiratory failure	1 (1.4)

a Adverse events assessed by the principal investigator as possibly, probably, or highly probably related to HSPPC-96 administration and with NCI CTC severity or investigator-determined severity of ⩾3.

b Patients are counted once within each body system and preferred term.

c This patient was in the treatment group that received HSPPC-96 only.

## References

[bib1] Alatrash G, Hutson TE, Molto L, Richmond A, Nemec C, Mekhail T, Elson P, Tannenbaum C, Olencki T, Finke J, Bukowski RM (2004) Clinical and immunologic effects of subcutaneously administered interleukin-12 and interferon alfa-2b: phase I trial of patients with metastatic renal cell carcinoma or malignant melanoma. J Clin Oncol 22: 2891–2900 15254058 10.1200/JCO.2004.10.045

[bib2] Artz AS, Van Besien K, Zimmerman T, Gajewski TF, Rini BI, Hu HS, Stadler WM, Vogelzang NJ (2004) Long-term follow-up of nonmyeloablative allogeneic stem cell transplantation for renal cell carcinoma: The University of Chicago Experience. Bone Marrow Transplant 35: 253–260 10.1038/sj.bmt.1704760 15543195

[bib3] Binder RJ, Blachere NE, Srivastava PK (2001) Heat shock protein-chaperoned peptides but not free peptides introduced into the cytosol are presented efficiently by major histocompatibility complex I molecules. J Biol Chem 276: 17163–17171 11278929 10.1074/jbc.M011547200

[bib4] Blachere NE, Udono H, Janetzki S, Li Z, Heike M, Srivastava PK (1993) Heat shock protein vaccines against cancer. J Immunother Emphasis Tumor Immunol 14: 352–356 8280719 10.1097/00002371-199311000-00016

[bib5] Childs R, Chernoff A, Contentin N, Bahceci E, Schrump D, Leitman S, Read EJ, Tisdale J, Dunbar C, Linehan WM, Young NS, Barrett AJ (2000) Regression of metastatic renal-cell carcinoma after nonmyeloablative allogeneic peripheral-blood stem-cell transplantation. N Engl J Med 343: 750–758 10984562 10.1056/NEJM200009143431101

[bib6] MRCRC Collaborators (1999) Interferon-alpha and survival in metastatic renal carcinoma: early results of a randomised controlled trial. Lancet 353: 14–17 10023944

[bib7] Elhilali MM, Gleave M, Fradet Y, Davis I, Venner P, Saad F, Klotz L, Moore R, Ernst S, Paton V (2000) Placebo-associated remissions in a multicentre, randomized, double-blind trial of interferon gamma-1b for the treatment of metastatic renal cell carcinoma. The Canadian Urologic Oncology Group. BJU Int 86: 613–618 11069364 10.1046/j.1464-410x.2000.00880.x

[bib8] Ernstoff MS, Crocenzi TS, Seigne JD, Crosby NA, Cole BF, Fisher JL, Uhlenhake JC, Mellinger D, Foster C, Farnham CJ, Mackay K, Szczepiorkowski ZM, Webber SM, Schned AR, Harris RD, Barth Jr RJ, Heaney JA, Noelle RJ (2007) Developing a rational tumor vaccine therapy for renal cell carcinoma: immune Yin and Yang. Clin Cancer Res 13: 733s–740s 17255302 10.1158/1078-0432.CCR-06-2064

[bib9] Escudier B, Eisen T, Stadler WM, Szczylik C, Oudard S, Siebels M, Negrier S, Chevreau C, Solska E, Desai AA, Rolland F, Demkow T, Hutson TE, Gore M, Freeman S, Schwartz B, Shan M, Simantov R, Bukowski RM, the T.S.G. (2007) Sorafenib in advanced clear-cell renal-cell carcinoma. N Engl J Med 356: 125–134 17215530 10.1056/NEJMoa060655

[bib10] Fyfe G, Fisher RI, Rosenberg SA, Sznol M, Parkinson DR, Louie AC (1995) Results of treatment of 255 patients with metastatic renal cell carcinoma who received high-dose recombinant interleukin-2 therapy. J Clin Oncol 13: 688–696 7884429 10.1200/JCO.1995.13.3.688

[bib11] Hainsworth JD, Sosman JA, Spigel DR, Edwards DL, Baughman C, Greco A (2005) Treatment of metastatic renal cell carcinoma with a combination of bevacizumab and erlotinib. J Clin Oncol 23: 7889–7896 16204015 10.1200/JCO.2005.01.8234

[bib12] Janetzki S, Palla D, Rosenhauer V, Lochs H, Lewis JJ, Srivastava PK (2000) Immunization of cancer patients with autologous cancer-derived heat shock protein gp96 preparations: a pilot study. Int J Cancer 88: 232–238 11004674 10.1002/1097-0215(20001015)88:2<232::aid-ijc14>3.0.co;2-8

[bib13] Mazzaferro V, Coppa J, Carrabba MG, Rivoltini L, Schiavo M, Regalia E, Mariani L, Camerini T, Marchiano A, Andreola S, Camerini R, Corsi M, Lewis JJ, Srivastava PK, Parmiani G (2003) Vaccination with autologous tumor-derived heat-shock protein Gp96 after liver resection for metastatic colorectal cancer. Clin Cancer Res 9: 3235–3245 12960108

[bib14] McDermott DF, Regan MM, Clark JI, Flaherty LE, Weiss GR, Logan TF, Kirkwood JM, Gordon MS, Sosman JA, Ernstoff MS, Tretter CP, Urba WJ, Smith JW, Margolin KA, Mier JW, Gollob JA, Dutcher JP, Atkins MB (2005) Randomized phase III trial of high-dose interleukin-2 *vs* subcutaneous interleukin-2 and interferon in patients with metastatic renal cell carcinoma. J Clin Oncol 23: 133–141 15625368 10.1200/JCO.2005.03.206

[bib15] Motzer RJ, Hutson TE, Tomczak P, Michaelson MD, Bukowski RM, Rixe O, Oudard S, Negrier S, Szczylik C, Kim ST, Chen I, Bycott PW, Baum CM, Figlin RA (2007) Sunitinib *vs* interferon alfa in metastatic renal-cell carcinoma. N Engl J Med 356: 115–124 17215529 10.1056/NEJMoa065044

[bib16] Motzer RJ, Mazumdar M, Bacik J, Berg W, Amsterdam A, Ferrara J (1999) Survival and prognostic stratification of 670 patients with advanced renal cell carcinoma. J Clin Oncol 17: 2530–2540 10561319 10.1200/JCO.1999.17.8.2530

[bib17] Palladino Jr MA, Srivastava PK, Oettgen HF, DeLeo AB (1987) Expression of a shared tumor-specific antigen by two chemically induced BALB/c sarcomas. Cancer Res 47: 5074–5079 3497717

[bib18] Pyrhonen S, Salminen E, Ruutu M, Lehtonen T, Nurmi M, Tammela T, Juusela H, Rintala E, Hietanen P, Kellokumpu-Lehtinen PL (1999) Prospective randomized trial of interferon alfa-2a plus vinblastine *vs* vinblastine alone in patients with advanced renal cell cancer. J Clin Oncol 17: 2859–2867 10561363 10.1200/JCO.1999.17.9.2859

[bib19] Rini BI, Halabi S, Barrier R, Margolin KA, Avigan D, Logan T, Stadler WM, McCarthy PL, Linker CA, Small EJ (2006) Adoptive immunotherapy by allogeneic stem cell transplantation for metastatic renal cell carcinoma: A CALGB intergroup phase II study. Biol Blood Marrow Transplant 12: 778–785 16785067 10.1016/j.bbmt.2006.03.011

[bib20] Rivoltini L, Castelli C, Carrabba M, Mazzaferro V, Pilla L, Huber V, Coppa J, Gallino G, Scheibenbogen C, Squarcina P, Cova A, Camerini R, Lewis JJ, Srivastava PK, Parmiani G (2003) Human tumor-derived heat shock protein 96 mediates *in vitro* activation and *in vivo* expansion of melanoma- and colon carcinoma-specific T cells. J Immunol 171: 3467–3474 14500642 10.4049/jimmunol.171.7.3467

[bib21] Srivastava PK, Das MR (1984) The serologically unique cell surface antigen of Zajdela ascitic hepatoma is also its tumor-associated transplantation antigen. Int J Cancer 33: 417–422 6698641 10.1002/ijc.2910330321

[bib22] Suto R, Srivastava PK (1995) A mechanism for the specific immunogenicity of heat shock protein-chaperoned peptides. Science 269: 1585–1588 7545313 10.1126/science.7545313

[bib23] Tannir NM, Cohen L, Wang X, Thall P, Mathew PF, Jonasch E, Siefker-Radtke A, Pagliaro LC, Ng CS, Logothetis C (2006) Improved tolerability and quality of life with maintained efficacy using twice-daily low-dose interferon-alpha-2b: results of a randomized phase II trial of low-dose *vs* intermediate-dose interferon-alpha-2b in patients with metastatic renal cell carcinoma. Cancer 107: 2254–2261 17029276 10.1002/cncr.22253

[bib24] Udono H, Levey DL, Srivastava PK (1994) Cellular requirements for tumor-specific immunity elicited by heat shock proteins: tumor rejection antigen gp96 primes cd8+ T cells *in vivo*. Proc Natl Acad Sci USA 91: 3077–3081 7909157 10.1073/pnas.91.8.3077 PMC43518

[bib25] Uemura H, Fujimoto K, Tanaka M, Yoshikawa M, Hirao Y, Uejima S, Yoshikawa K, Itoh K (2006) A phase I trial of vaccination of CA9-derived peptides for HLA-A24-positive patients with cytokine-refractory metastatic renal cell carcinoma. Clin Cancer Res 12: 1768–1775 16551861 10.1158/1078-0432.CCR-05-2253

[bib26] Yang JC, Haworth L, Sherry RM, Hwu P, Schwartzentruber DJ, Topalian SL, Steinberg SM, Chen HX, Rosenberg SA (2003a) A randomized trial of bevacizumab, an anti-vascular endothelial growth factor antibody, for metastatic renal cancer. N Engl J Med 349: 427–434 12890841 10.1056/NEJMoa021491 PMC2275324

[bib27] Yang JC, Sherry RM, Steinberg SM, Topalian SL, Schwartzentruber DJ, Hwu P, Seipp CA, Rogers-Freezer L, Morton KE, White DE, Liewehr DJ, Merino MJ, Rosenberg SA (2003b) Randomized study of high-dose and low-dose interleukin-2 in patients with metastatic renal cancer. J Clin Oncol 21: 3127–3132 12915604 10.1200/JCO.2003.02.122 PMC2275327

